# A Comparative Study between Single-Level Oblique Lumbar Interbody Fusion with Transforaminal Lumbar Interbody Fusion for Lumbar Adjacent Segment Disease

**DOI:** 10.3390/jcm13195843

**Published:** 2024-09-30

**Authors:** Chung-Tse Chang, Yu-Hsien Lin, Yun-Che Wu, Cheng-Min Shih, Kun-Hui Chen, Chien-Chou Pan, Cheng-Hung Lee

**Affiliations:** 1 Department of Orthopedics, Taichung Veterans General Hospital, Taichung 40705, Taiwan; ely83310@gmail.com (C.-T.C.); adonisvgh@gmail.com (C.-C.P.); 2 Department of Physical Therapy, Hungkuang University, Taichung 43304, Taiwan; 3 College of Medicine, National Chung Hsing University, Taichung 40227, Taiwan; 4 Department of Biomedical Engineering, College of Intelligent Technology, Hungkuang University, Taichung 43304, Taiwan; 5 Department of Rehabilitation Science, Jenteh Junior College of Medicine, Nursing and Management, Miaoli 35664, Taiwan

**Keywords:** lumbar spine, adjacent segment disease (ASD), oblique lumbar interbody fusion (OLIF), transforaminal lumbar interbody fusion (TLIF)

## Abstract

**Background/Objectives**: Various surgical approaches have been proposed for treating adjacent segment disease (ASD) after lumbar fusion. However, studies using oblique lumbar interbody fusion (OLIF) to treat ASD are lacking. The current study assessed the postoperative outcomes of single-level OLIF for ASD, comparing the results with those for patients undergoing transforaminal lumbar interbody fusion (TLIF). **Methods**: Patients who underwent single-level OLIF or TLIF for lumbar ASD were retrospectively included. Clinical outcomes, that is, the results of assessments using the Euroqol 5-Dimension quality of life scale (EQ-5D), the Oswestry Disability Index, and the visual analog scale, were evaluated. Radiologic parameters, including disc height (DH), segmental lordosis (SL), segmental coronal angle (SCA), lumbar lordosis, and pelvic incidence–lumbar lordosis mismatch, were also assessed. **Results**: A total of 65 patients were enrolled: 32 in the OLIF group and 33 in the TLIF group. The median follow-up time was 24.0 months in both groups. The clinical outcomes and radiologic parameters significantly improved in both groups postoperatively. According to intergroup comparisons, the OLIF group had significantly less blood loss and superior improvement in radiologic parameters (DH, SL, and SCA) whereas the TLIF group had significantly shorter operation times. For the OLIF patients who did not undergo posterior decompression, the operation time was similar to that of the TLIF group, but the surgical blood loss and length of hospital stay were significantly reduced compared with the TLIF group. **Conclusions**: Compared with TLIF, OLIF provides similar clinical outcomes, leads to less surgical blood loss, and has superior radiologic parameters; however, the operation time is significantly longer. OLIF without posterior decompression may be a superior option to TLIF for certain patients.

## 1. Introduction

Adjacent segment disease (ASD) is a common long-term complication following lumbar fusion. ASD is associated with various radiographic findings, such as disc degeneration, facet hypertrophy, instability, and spondylolisthesis [1]. Studies have indicated that approximately 70% of patients exhibit signs of ASD on radiographic images post-lumbar fusion surgery, with 30% of these patients becoming symptomatic and requiring further intervention [2,3].

Numerous surgical approaches have been proposed for treating ASD [4,5,6,7,8,9]. The conventional posterior approach with decompression and extended fusion has long been the standard treatment because of its satisfactory outcomes [7,9]. However, posterior revision surgery can be technically challenging because of scar tissue that forms after the previous surgery. Additionally, the posterior approach may cause secondary damage to the paraspinal muscles and soft tissue, leading to postoperative back pain, extensive blood loss, and prolonged recovery [10].

Since oblique lumbar interbody fusion (OLIF) was introduced by Mayer et al. in 1997 [11], the minimally invasive technique has been widely used for treating degenerative lumbar disc disease. In OLIF, the disc space is accessed through an anterolateral retroperitoneal approach, which avoids the challenges associated with posterior surgery such as incidental durotomy and postoperative muscle weakness [5,12]. However, few studies have reported on the outcomes of OLIF used to treat ASD.

This study retrospectively investigated the clinical and radiographic outcomes of single-level OLIF for treating symptomatic lumbar ASD and compared them with those of patients who underwent transforaminal lumbar interbody fusion (TLIF) for ASD.

## 2. Materials and Methods

### 2.1. Study Population

This retrospective case–control study was conducted at a single institution and was approved by the study institution’s institutional review board (CE22433A). We reviewed patients who underwent single-level revisional OLIF or TLIF for symptomatic lumbar ASD between April 2016 and December 2022. The inclusion criteria were (1) undergoing single-level OLIF or TLIF for treatment of lumbar ASD after failed conservative treatment; (2) having X-ray or magnetic resonance images (MRI) revealing features of ASD such as disc herniation, spinal stenosis, and spondylolisthesis at the adjacent segment; (3) having primary lumbar fusion for degenerative disease; (4) having no prior instrumentation/procedure at the index surgery level; and (5) undergoing at least a 12-month follow-up after surgery. Symptomatic ASD was diagnosed on the basis of the presence of clinical symptoms compatible with radiologic evidence of ASD. The exclusion criteria were (1) undergoing primary surgery for nondegenerative diseases such as trauma, tumor, or infection; (2) undergoing less than 12 months of follow-up; and (3) undergoing standalone OLIF or OLIF with anterolateral screw/plate fixation.

**Figure 1:**
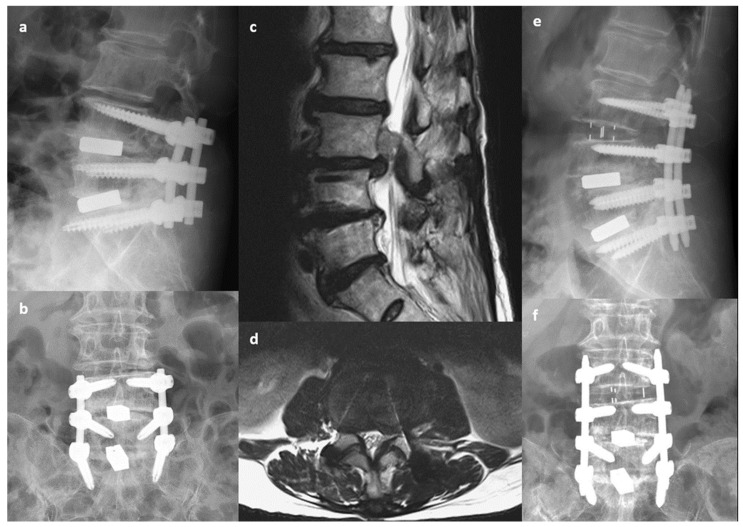
A 78-year-old woman with low back pain and right thigh radiation. Primary transforaminal lumbar interbody fusion was performed 3 years previously to treat spinal stenosis and disc herniation at L4-5-S1. (**a**,**b**) Preoperative X-ray indicating decreased disc height at L3-4. (**c**,**d**) Preoperative MRI indicating uncontained disc extrusion with upper migration at L3-4. (**e**,**f**) Postoperative X-ray indicating oblique lumbar interbody fusion at L3-4 with extension of posterior instrumentation to L3. Left-side laminotomy of L3 was performed to remove the migrated disc.

**Figure 2:**
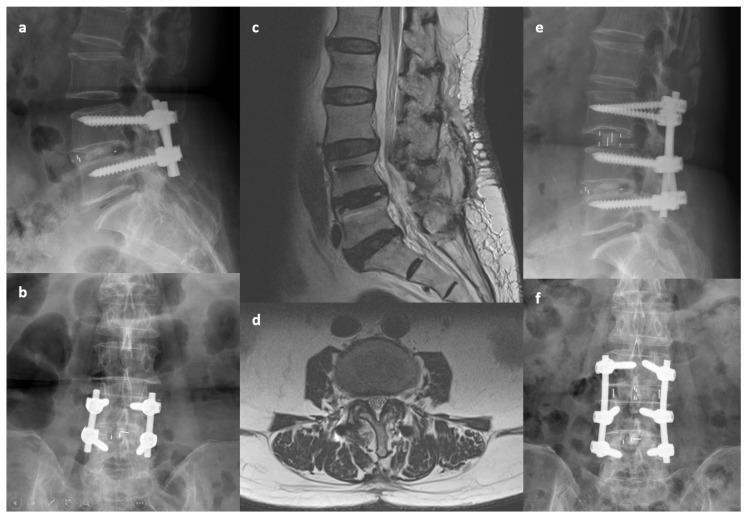
An 81-year-old woman presented with low back pain, left thigh radiation, and claudication for 9 months. She had received transforaminal lumbar interbody fusion performed at the L4-5 segment 7 years previously for spondylolisthesis and spinal stenosis. (**a**,**b**) Preoperative X-ray images. (**c**,**d**) Preoperative MRI indicating central canal stenosis at L3-4. (**e**,**f**) Postoperative X-ray indicating oblique lumbar interbody fusion at L3-4 with extension of posterior instrumentation. The lower endplate of L3 may have been damaged during surgery.

After the preliminary screening, 72 consecutive patients were enrolled. Seven of these patients were excluded: five because they had an inadequate postoperative follow-up period, one because they were undergoing standalone OLIF, and one because they were undergoing OLIF with anterolateral screw fixation. The remaining 65 patients were segmented into 2 groups, with 32 in the OLIF group and 33 in the TLIF group. All patients in the OLIF group received posterior pedicle screw fixation with or without posterior decompression. The criteria for additional posterior decompression in the OLIF group were having noncontained disc herniation (disc extrusion or sequestration), having severe lumbar canal stenosis (Schizas grade C-D) [13] with locked or “autofusion” facets, and having stenosis due to bony compression. A total of 18 patients received posterior decompression (OLIF + D), and 14 patients did not (OLIF − D); two of the cases are illustrated in Figure 1 and Figure 2. Patient demographic and perioperative data obtained from medical records were reviewed. Demographic data, including age, sex, body mass index (BMI), American Society of Anesthesiology (ASA) classification, comorbidities, level of ASD, interval from the index surgery to the previous fusion surgery, number of initial fusion segments, number of previous lumbar surgeries, and follow-up time, were collected (Table 1). Data on perioperative variables such as operation time, blood loss, length of hospital stay, cage type/height, skin incision, cage subsidence, reoperation, and perioperative complications were also collected (Table 2). Reoperation was defined as any additional surgery performed at or adjacent to the index level within 1 year after the index surgery. Patients were selected for OLIF versus TLIF on the basis of the surgeon’s clinical decision and the patient’s vascular anatomy.

### 2.2. Surgical Technique

#### 2.2.1. OLIF Group

The patient was positioned in the right lateral decubitus position. The center of the target disc, inferior endplate of the upper vertebra, and superior endplate of the lower vertebra were marked under fluoroscopy. A 4 cm skin incision was made 5 cm anterior to the center of the target disc. The external oblique, internal oblique, and transverse abdominal muscles were dissected and split. The peritoneal content was moved anteriorly to expose the retroperitoneal space, and the psoas muscle was retracted posteriorly to access the intervertebral disc. Following discectomy, a 6° lordotic polyetheretherketone (PEEK) cage (Clydesdale Spinal System; Medtronic, Minneapolis, MN, USA) filled with synthetic bone graft was inserted. The cage position was verified under fluoroscopy, after which the wound was closed.

The patient was subsequently repositioned in the prone position. A skin incision was made and extended along the previous scar (midline or Wiltse). Posterior instrumentation was extended to the ASD level with pedicle screws, which were inserted using the open technique. The old pedicle screws were replaced with larger diameter screws if the old screws were loosened or did not fit the new rod. For additional posterior decompression, partial disease-level laminectomy was performed, the ligamentum flavum was excised, and the lateral recess and foramen were decompressed until the nerve root and lateral border of the dura were freely movable.

A Hemovac drain was placed in the posterior wound and removed between 2 and 3 days after surgery if drainage was less than 50 mL. Intravenous and oral pain medications, such as morphine and nonsteroidal anti-inflammatory drugs (NSAIDs), were administered as necessary. Patients began bearing weight 2 days after surgery and wore a lumbar brace for 3 months.

#### 2.2.2. TLIF Group

With the patient in the prone position, a skin incision was made along the old scar to expose the index level and previous implant. A partial facetectomy and laminectomy were performed, decompressing the lateral recess and foramen until the exiting root and transverse root were visible and free of compression. After disc space preparation, TLIF was performed with a single cage filled with autogenous or synthetic bone grafts. Pedicle screws were inserted using the standard open technique. The management of the posterior instrumentation for the TLIF group was the same as that in the OLIF group.

As in the OLIF group, a Hemovac drain was placed in the posterior wound and removed between 2 and 3 days after surgery if drainage was less than 50 mL. Intravenous and oral pain medications, such as morphine and NSAIDs, were administered as necessary. Patients began bearing weight 2 days after surgery and wore a lumbar brace for 3 months.

### 2.3. Clinical Evaluation

Clinical outcomes were assessed using patient-reported outcome measures, such as the Oswestry Disability Index (ODI), Euroqol 5-Dimensions (EQ-5D) quality of life scale, and the visual analog scale (VAS) for back and leg pain. All clinical scores were recorded preoperatively, immediately postoperatively, 3 months postoperatively, and at the last follow-up using records from the outpatient department or phone interviews.

A clinically relevant successful treatment for OLIF or TLIF was defined as one that achieved a predetermined cutoff value for minimal clinically important difference (MCID). The specific MCID values and the proportion of patients achieving MCID are detailed in the supplementary file (Appendix A).

### 2.4. Radiographic Evaluation

Preoperative spine MRIs were evaluated for all patients to assist in diagnosis and surgical planning, and the radiographic features of ASD were recorded. Disc height (DH), segmental lordosis (SL), segmental coronal angle (SCA), lumbar lordosis (LL), and pelvic incidence and LL mismatch (PI-LL mismatch) were measured on plain anterior–posterior and lateral radiographs, and the differences between preoperative and postoperative measurements were calculated. DH was calculated as the average measure of four locations (anteriorly/posteriorly on lateral views and right/left on anterior–posterior views). SL, SCA, and LL were measured using Cobb’s method. The fusion status of the index level was evaluated using the criteria of Santos et al. [14] at the last follow-up as follows: (1) Grade I: no fusion with motion or radiolucency around the device, (2) Grade II: partial fusion with no motion around the device and no definite bony opacity formation in or around the cage, and (3) Grade III: complete fusion with no motion or radiolucency around the device and definite bony opacity formation in or around the cage. Successful fusion was defined as Grade II or Grade III in the study cohorts. Cage subsidence was classified on lateral radiographs using the following scale: Grade 0, between 0% and 24% loss of postoperative disc height; Grade I, between 25% and 49% loss of postoperative DH; Grade II, between 50% and 74% loss of postoperative DH; and Grade III, between 75% and 100% loss of postoperative DH. Radiographic parameters were measured using a digital imaging system (Surgimap^®^; Nemaris Inc., New York, NY, USA).

### 2.5. Statistical Analysis

Continuous variables are presented as medians and interquartile ranges (IQRs), and categorical variables are presented as numbers and percentages. The chi-square test or Fisher’s exact test, the Mann–Whitney U test, and the Kruskal–Wallis test were used to compare the demographic data, perioperative variables, clinical scores, and radiographic parameters between groups. The Wilcoxon signed-rank test was used to compare the pre- and post-operative radiographic parameters, and Friedman tests were used to compare the pre- and post-operative clinical scores. A post hoc analysis (Dunn–Bonferroni) was conducted when differences between the groups were significant. SPSS version 27.0 (International Business Machines, Armonk, NY, USA) was used for all statistical analyses. Significance was set at *p* < 0.05. Statistical power was calculated using post hoc analysis, and the results are detailed in the supplementary file (Appendix A).

## 3. Results

### 3.1. Demographic Data

Among the 32 patients in the OLIF group, the median age was 68 years (IQR 59.0–74.0), and 25 (78.1%) were women. The median BMI was 26.8 kg/m^2^ (IQR 24.3–29.7). The levels of ASD were as follows: 2 patients (6.3%) at L1-2, 9 (28.1%) at L2-3, 14 (43.8%) at L3-4, 7 (21.9%) at L4-5, and 0 at L5-S1. Thirteen patients (40.6%) had one previous fusion segment, fourteen (43.8%) had two, and five (15.6%) had three. Twenty-eight patients (87.5%) had one previous lumbar surgery, three (9.4%) had two, and one (3.1%) had three. The ASD level was cephalad to the old fusion segment in 29 patients (90.6%). Eighteen patients (56.3%) underwent additional posterior decompression. The OLIF group did not significantly differ from the TLIF group in terms of sex, age, BMI, ASA classification, smoking status, comorbidities, follow-up time, number of previous fused segments, number of previous lumbar surgeries, or interval from previous fusion to index surgery (Table 1).

Regarding operative variables (Table 2) in the TLIF group, 25 PEEK cages (81.8%), 5 porous tantalum cages (15.1%), and 1 carbon fiber cage (3.0%) were used. Most patients received a midline posterior skin incision (*n* = 32, 97%). In the OLIF group, all patients (n = 32, 100%) received PEEK cages; 27 patients (84.4%) received a midline incision for posterior approach, and 5 (15.6%) received a Wiltse incision. The estimated blood loss was significantly lower in the OLIF group (325.0 mL, IQR 231.3–500.0) than in the TLIF group (600.0 mL, IQR 450.0–765.0, *p* < 0.001). However, the operation time was significantly longer in the OLIF group (327.5 min, IQR 292.5–377.5) than in the TLIF group (300.0 min, IQR 277.5–320.0, *p* = 0.039). The rate of cage subsidence was 15.6% (n = 5) in the OLIF group and 15.2% (n = 5) in the TLIF group. All instances of subsidence were grade 0, with the exception of one instance of grade 1 in the TLIF group. No significant difference was observed between the groups regarding cage height, posterior skin incision, length of hospital stay, subsidence rate, complication rate, and reoperation rate (Table 2).

### 3.2. Clinical and Radiological Outcomes

The clinical scores in both groups significantly improved at 3 months postoperation and at the last follow-up relative to before the operation. Group comparisons of the ODI score, EQ-5D score, and VAS score for back and leg pain revealed no significant difference at any time point between the OLIF and TLIF groups (Table 3).

Regarding radiographic parameters, postoperative DH, SL, and SCA improved significantly in the OLIF group (Table 4), whereas LL and PI-LL mismatch did not. In the TLIF group, only DH exhibited significant postoperative improvement. Intergroup comparisons revealed that the fusion rates for the groups were similar. However, the DH at 1 year postoperation (OLIF 8.8 mm, IQR 7.7–9.9 vs. TLIF 7.1 mm, IQR 6.1–7.7, *p* < 0.001) significantly improved in the OLIF group. The differences in DH, SL, and SCA between the preoperative measurements and those at the 1-year follow-up were significantly larger in the OLIF group.

### 3.3. OLIF with (OLIF + D) and without Posterior Decompression (OLIF − D) versus TLIF Group

According to the comparison of the subgroup outcomes between the OLIF + D, OLIF − D, and TLIF groups (Table 5), the OLIF − D group had significantly shorter hospital stays (6.0 days, IQR 5.0–6.0) than the OLIF + D and TLIF groups, whereas the OLIF + D group had significantly longer operation times (362.0 min, IQR 315.0–401.3). Blood loss was greater in the TLIF group (600.0 mL, IQR 450.0–765.0), but no significant difference was observed between the OLIF − D and OLIF + D groups. Regarding cage size, the OLIF − D group had significantly greater cage heights (12.0 mm, IQR 10.0–12.0); no difference in cage height was observed between the OLIF + D and TLIF groups. The subsidence rate, reoperation rate, and clinical scores did not statistically differ between the groups.

### 3.4. Complications and Reoperations

Five perioperative complications occurred in the OLIF group (5/32, 15.6%), that is, three cases of postoperative ileus (3/32, 9.3%) that subsided with supportive care, one case of postoperative leg pain (1/32, 3.1%) that resolved completely at 1 month following the operation, and one case of postoperative pulmonary edema (1/32, 3.1%) that improved with medical treatment. In the TLIF group, four perioperative complications were recorded (4/33, 12.1%), that is, two dural tears (2/33, 6.0%) that were repaired intraoperatively, one surgical site infection (1/33, 3.0%) that was successfully managed with antibiotics, and one case of postoperative ileus (1/33, 3.1%).

Three reoperations in the OLIF group (9.4%) and two in the TLIF group (6.1%) were required. In the OLIF group, one patient received reoperation because of ASD progression next to the index level, and one patient received reoperation because of pedicle screw breakage at the ASD level 11 months after the index surgery. Another patient with a previously fused L4-5 segment had an index surgery at L3-4 with posterior decompression. For this patient, radiculopathy persisted several months after OLIF; MRIs revealed residual L4-5 lateral recess stenosis and an L4-5 open laminectomy was performed. In the TLIF group, one patient received reoperation due to ASD progression next to the index level, and another patient experienced a traumatic event 9 months after the operation that resulted in an epidural hematoma and nerve compression at the index level requiring evacuation through reoperation.

## 4. Discussion

Since the introduction of OLIF in 1997 [11], the technique has been widely used to treat various conditions, including ASD. Some experts have advocated for primary OLIF as a minimally invasive surgery that leads to positive clinical results, shorter operating times, low blood loss, and rapid recovery [12,15]. However, the difficulties encountered when performing OLIF for revision surgery, such as instrumentation revision and posterior decompression, are similar to those encountered during posterior revision surgery. Therefore, the result of OLIF in treating ASD may vary, and the outcome may still be elusive.

Two types of OLIF are available for treating lumbar ASD: standalone OLIF and OLIF with screw instrumentation. Studies have indicated that standalone OLIF achieves satisfactory clinical results with shorter operating times and less blood loss than TLIF in treating ASD [4,5,8]. However, reoperation and mechanical stability remain concerns associated with OLIF. Endplate damage during the procedure is a common complication and may cause cage subsidence [16]. Biomechanical studies have demonstrated that a standalone OLIF cage imposes greater stress on the endplate than OLIF with pedicle screw fixation does [17,18], especially in osteoporotic bone, which is common among those with degenerative spine diseases.

Clinical reports have highlighted the issues associated with standalone OLIF. For example, Aichmair et al. reported a reoperation rate of 25.8% (8/31 cases) after standalone OLIF for ASD, which was higher than that for OLIF with circumferential fusion (14.3%, 3/21) [4]. Additionally, Jin et al. reported a reoperation rate of 8.3% (1/12 cases) after OLIF (75% of cases were standalone) due to cage subsidence [5], and Ohtori et al. reported a reoperation rate of 9.5% for standalone OLIF [19]. Therefore, standalone OLIF for lumbar ASD may entail a higher risk of instability and revision. Consequently, the procedures for all patients receiving OLIF in the present study were supplemented with bilateral pedicle screw fixation.

Most of the demographic data for the OLIF and TLIF groups were similar (Table 1), with the exception of the level of ASD. This may be because four patients in the TLIF group had an index surgical level at the L5-S1 segment, whereas none in the OLIF group did. The surgical corridor for performing OLIF at the L5-S1 segment is limited by vascular anatomy [20], rendering the approach difficult. Although the use of the OLIF procedure to access the L5-S1 segment has yielded satisfactory outcomes [21,22], the procedure is technically challenging and necessitates meticulous preoperative planning and patient selection. Under such circumstances, TLIF or anterior lumbar interbody fusion (ALIF) [23] are alternative treatments for ASD at the L5-S1 segment.

The clinical scores of the OLIF and TLIF groups did not differ significantly over time. However, radiologic findings indicated superior outcomes in the OLIF group for several parameters, including postoperative 1-year DH (8.8 mm, IQR 7.9–9.9), change in DH (2.8 mm, IQR 2.0–5.0), SL (3.7 mm, IQR 1.5–5.5), and SCA (−1.3°, IQR −2.5° to −0.5°) between the preoperative measurements and those of the 1-year follow-up. This result suggests that OLIF resulted in superior local alignment correction due to the insertion of a 6° lordotic cage and superior mechanical stability due to the larger area of the OLIF cage. By contrast, the TLIF approach’s spatial limitations required the insertion of a smaller cage without a lordotic curve, leading to inferior radiologic outcomes. Nonetheless, the superior local alignment correction in the OLIF group did not always translate into superior patient-reported outcomes. Furthermore, no difference in fusion rates or global sagittal alignments, such as LL or PI-LL mismatch, was observed between the two groups. Previous studies have suggested that achieving balanced sagittal alignment leads to less pain and improved quality of life [24,25,26]. However, the local alignment correction achieved with single-level OLIF may not substantially influence global alignment. Therefore, future studies are warranted to investigate multilevel OLIF for lumbar ASD.

Regarding perioperative data, the OLIF group’s operation time was significantly longer than that of the TLIF group (327.5 min, IQR 292.5–377.5 vs. 300.0 min, IQR 277.5–320.0, *p* = 0.039). Two factors contributed to this difference. First, all patients who underwent OLIF received bilateral pedicle screws, and 56.3% (18/32) underwent posterior decompression, adding complexity to the surgery. The difficulties encountered during posterior laminectomy and laminotomy in the patients undergoing OLIF were similar to those encountered in the patients in the TLIF group, partially offsetting the minimally invasive advantage of OLIF. Second, the requirement for repositioning the patients from the lateral decubitus to the prone position after the anterolateral procedure further complicated the surgery, leading to a need for a second round of prepping, draping, and room positioning that extended the operation time by between 45 and 60 min [27,28]. Single-position anterior–posterior fusion surgery has been suggested to mitigate the challenges of repositioning [28,29], indicating a future direction for OLIF treatment for ASD.

The present study observed significantly less blood loss in the OLIF group (325.0 mL, IQR 231.3–500.0) than in the TLIF group (600.0 mL, IQR 450.0–765.0, *p* < 0.001). The presence of 14 patients in the OLIF group (43.7%) who did not receive posterior decompression and the performance of discectomy and cage insertion from a posterior approach in the TLIF group likely contributed to the significantly increased blood loss observed in the TLIF group. Furthermore, the spinal canal, dura, and nerve roots are often surrounded by an abundant vertebral venous plexus [30]; radicular arteries branch off from segmental arteries and enter the dura through the neuroforamen [31], making bleeding inevitable during posterior decompression, nerve root retraction, and disc space preparation.

Among the patients undergoing OLIF in the present study, the indications for additional posterior decompression included noncontained disc herniation, severe lumbar canal stenosis (Schizas grade C-D), and locked or “autofusion” facets that could not be spread open during cage insertion. These indications for posterior decompression led us to divide the OLIF patients into two subgroups: OLIF + D and OLIF − D. The clinical outcomes were similar for both the OLIF groups and the TLIF group (Table 5). However, although the operation time was comparable between the OLIF − D and TLIF groups, the operation time was longer for the OLIF + D group. Notably, blood loss was significantly reduced in the OLIF − D group relative to the TLIF group. Additionally, the OLIF − D group had significantly shorter hospital stays than the OLIF + D and TLIF groups. These results suggest that OLIF without posterior decompression may be a superior option for some patients. The patients who underwent OLIF − D typically had less severe degenerative ASD (e.g., Schizas grade B spinal canal stenosis), greater DH, and less bony spur formation and were less likely to have a stiff or fused ASD segment, enabling the insertion of a larger cage (OLIF − D 12.0 mm, IQR 10.0–12.0). For the OLIF + D group, the advantage over TLIF was less pronounced.

The primary strength of this study is its presentation of the outcomes of OLIF for ASD. All of our OLIF patients received posterior instrumentation with or without posterior decompression. By contrast, previous studies have primarily employed standalone OLIF [4,5,8,19]. Although standalone OLIF obviates the need for posterior decompression, revision of previous implants, and patient repositioning—resulting in faster operating times, less blood loss, and shorter hospital stays compared with our OLIF patients—our approach provides a more comprehensive evaluation by including posterior instrumentation and decompression. These findings provide insights for surgeons choosing between OLIF and TLIF for their patients.

This study has several limitations. First, the study had a retrospective design, with surgeries performed by multiple surgeons, which may have introduced case selection bias. Second, the fusion rate was determined using radiography rather than computed tomography. Third, only single-level interbody fusion was conducted, leaving the results for multilevel ASD uncertain. Fourth, the precise indication for additional posterior decompression in patients undergoing OLIF remains unclear, and our selection criteria may not be universally accepted. Finally, the study had a short follow-up duration and a relatively small sample size, which may not fully represent the outcomes of the surgery for ASD. Therefore, larger prospective studies with long-term comparisons with other surgical methods are required.

In conclusion, the OLIF and TLIF surgical techniques for treating ASD have distinct advantages and disadvantages. TLIF enables a shorter operation time, avoids repositioning, and does not require a second surgical incision. However, TLIF offers a limited capacity for alignment correction and results in more surgical blood loss. Additionally, nerve root retraction and cage insertion in TLIF may increase the potential for nerve injury. Despite the groups having similar overall complication rates, the TLIF group experienced two cases of dural tears (2/33, 6.0%), whereas the OLIF group experienced none. The requirement for posterior decompression in OLIF may significantly reduce its advantages over TLIF. Therefore, OLIF without posterior decompression is suitable for patients with favorable vascular anatomy, less severe spinal stenosis (Schizas grade B), or primary symptoms of spinal instability with minimal root sign (which precludes the requirement for posterior decompression), and a requirement for alignment correction (degenerative scoliosis or kyphosis). By contrast, TLIF may be a superior option for patients with more severe spinal stenosis (Schizas grade C-D, severe intracanal/intraforaminal bony spurs with locked facet joint), symptomatic ASD at the L5-S1 segment, previous abdominal surgery compromising the anterolateral approach to the lumbar spine, or those who cannot endure long operations under anesthesia, such as older individuals or those with multiple comorbidities.

## Figures and Tables

**Table 1:** Demographic features of the OLIF and TLIF groups.

	OLIF Group (n = 32)	TLIF Group (n = 33)	*p*-Value
Age, years	68.0 (59.0, 74.0)	71.0 (66.0, 76.5)	0.242
Female	25 (78.1%)	22 (66.7%)	0.302
BMI, kg/m^2^	26.8 (24.3, 29.7)	26.8 (24.6, 30.8)	0.646
**ASA classification**			0.899
1	1 (3.1%)	0 (0.0%)	
2	17 (53.1%)	19 (57.6%)	
3	14 (43.8%)	14 (42.4%)	
4	0 (0.0%)	0 (0.0%)	
5	0 (0.0%)	0 (0.0%)	
Smoker	5 (15.6%)	3 (9.1%)	0.475
**Comorbidity**			
Diabetes Mellitus	10 (31.3%)	12 (36.4%)	0.663
Cardiovascular disease	6 (18.8%)	10 (30.3%)	0.280
Chronic kidney disease	7 (21.9%)	6 (18.2%)	0.710
Pulmonary disease	5 (15.6%)	2 (6.1%)	0.258
**Number of previous fused segments**			0.496
1	13 (40.6%)	15 (45.5%)	
2	14 (43.8%)	16 (48.5%)	
3	5 (15.6%)	2 (6.1%)	
**Number of previous lumbar surgeries**			0.475
1	28 (87.5%)	25 (75.8%)	
2	3 (9.4%)	6 (18.2%)	
3	1 (3.1%)	2 (6.0%)	
**Type** **of previous lumbar** **fusion**			-
TLIF	19 (59.4%)	20 (60.6%)	
PLIF	4 (12.5%)	5 (15.2%)	
ALIF	3 (9.4%)	2 (6.1%)	
OLIF	2 (6.3%)	0 (0.0%)	
PLF	4 (12.5%)	6 (18.2%)	
**Level of ASD**			0.036 *
L1-2	2 (6.3%)	1 (3.0%)	
L2-3	9 (28.1%)	16 (48.5%)	
L3-4	14 (43.8%)	10 (30.3%)	
L4-5	7 (21.9%)	2 (6.1%)	
L5-S1	0 (0.0%)	4 (12.1%)	
Cephalad	29 (90.6%)	29 (87.9%)	1.000
**Radiologic presentation**			-
Herniated disc	9 (28.1%)	14 (42.4%)	
Spinal stenosis	27 (84.4%)	28 (84.8%)	
Spondylolisthesis	14 (43.8%)	9 (27.3%)	
Segmental kyphosis	1 (3.1%)	2 (6.0%)	
Interval ^†^, years	8.5 (3.8, 11.0)	6.0 (4.0, 8.5)	0.163
Follow-up time, months	24.0 (12.0, 24.0)	24.0 (12.0, 24.0)	0.849

^†^ Interval between index surgery and previous fusion surgery; * *p* < 0.05. OLIF, oblique lumbar interbody fusion; TLIF, transforaminal lumbar interbody fusion; PLIF, posterior lumbar interbody fusion; ALIF, anterior lumbar interbody fusion; PLF, posterolateral fusion.

**Table 2:** Perioperative data for the OLIF and TLIF groups.

	OLIF Group (n = 32)	TLIF Group (n = 33)	*p*-Value
**Cage type**			-
PEEK	32 (100%)	27 (81.8%)	
Porous tantalum	0 (0.0%)	5 (15.1%)	
Carbon fiber	0 (0.0%)	1 (3.0%)	
Cage height, mm	10.0 (8.0, 12.0)	10.0 (8.0, 10.0)	0.174
**Posterior skin incision**			0.105
Midline	27 (84.4%)	32 (97.0%)	
Wiltse	5 (15.6%)	1 (3.0%)	
Hospital stay, days	6.0 (6.0, 7.0)	7.0 (6.0, 8.0)	0.172
Operating time, mins	327.5 (292.5, 377.5)	300.0 (277.5, 320.0)	0.039 *
Blood loss, mL	325.0 (231.3, 500.0)	600.0 (450.0, 765.0)	<0.001 **
Posterior decompression	18 (56.3%)	33 (100.0%)	-
Subsidence	5 (15.6%)	5 (15.2%)	1.000
Perioperative complications	5 (15.6%)	4 (12.1%)	0.733
Reoperation	3 (9.4%)	2 (6.1%)	0.672

OLIF, oblique lumbar interbody fusion; TLIF, transforaminal lumbar interbody fusion; PEEK, polyetheretherketone, * *p* <.05, ** *p* < 0.01.

**Table 3:** Clinical outcomes for the OLIF and TLIF groups.

	OLIF Group (n = 32)	TLIF Group (n = 33)	*p*-Value
**VAS (back pain)**			
Preoperative	8.0 (7.0, 8.0)	8.0 (5.0, 10.0)	0.793
Postoperative 3 months	3.0 (0.3, 4.8)	3.0 (1.5, 4.0)	0.941
Last follow-up	3.0 (0.0, 4.8)	3.0 (2.0, 4.0)	0.640
Δ† (Last − Pre)	−4.5 (−6.0, −3.0)	−5.0 (−6.0, −1.5)	0.726
*p*-value (Pre vs. Last)	<0.01 **	<0.01 **	
**VAS (leg pain)**			
Preoperative	8.0 (6.3, 8.0)	8.0 (8.0, 10.0)	0.067
Postoperative 3 months	2.0 (0.0, 4.8)	3.0 (0.0, 3.5)	0.482
Last follow-up	0.0 (0.0, 3.0)	2.0 (0.0, 4.0)	0.303
Δ† (Last − Pre)	−4.5 (−7.8, −2.3)	−5.0 (−8.0, −3.5)	0.287
*p*-value (Pre vs. Last)	<0.01 **	<0.01 **	
**Oswestry Disability Index (ODI)**			
Preoperative	62.2 (53.3, 66.7)	60.0 (52.2, 67.8)	0.640
Postoperative 3 months	44.4 (37.8, 53.3)	40.0 (34.4, 53.3)	0.457
Last follow-up	42.2 (28.9, 55.6)	40.0 (30.0, 50.0)	0.337
Δ† (Last − Pre)	−20.0 (−30.0, −5.0)	−22.2 (−30.0, −10.0)	0.655
*p*-value (Pre vs. Last)	<0.01 **	<0.01 **	
**EQ5D**			
Preoperative	0.36 (0.36, 0.36)	0.36 (0.36, 0.36)	0.371
Postoperative 3 months	0.60 (0.54, 0.68)	0.60 (0.54, 0.63)	0.660
Last follow-up	0.65 (0.54, 0.73)	0.59 (0.54, 0.71)	0.490
Δ† (Last − Pre)	0.27 (0.18, 0.36)	0.24 (0.18, 0.36)	0.816
*p*-value (Pre vs. Last)	<0.01 **	<0.01 **	

† Difference between “Preoperative” and “Last follow-up”. ** *p* < 0.01. OLIF, oblique lumbar interbody fusion; TLIF, transforaminal lumbar interbody fusion.

**Table 4:** Radiologic outcomes for the OLIF and TLIF groups.

	OLIF Group (n = 32)	TLIF Group (n = 33)	*p*-Value
**Disc height (DH), mm**			
Preoperative	4.8 (3.9, 7.2)	5.0 (4.0, 6.0)	0.529
Postoperative 1 year	8.8 (7.7, 9.9)	7.1 (6.1, 7.7)	<0.001 **
Δ† (Post − Pre)	2.8 (2.0, 5.0)	2.0 (0.4, 3.5)	0.011 *
*p*-value (Pre vs. Post)	<0.001 **	<0.001 **	
**Segmental lordosis (SL), °**			
Preoperative	4.9 (2.4, 7.6)	5.6 (2.2, 9.9)	0.665
Postoperative 1 year	8.5 (7.3, 11.2)	7.0 (4.8, 10.2)	0.065
Δ† (Post − Pre)	3.7 (1.5, 5.5)	1.2 (−1.4, 4.3)	0.012 *
*p*-value (Pre vs. Post)	<0.001 **	0.059	
**Segmental coronal angle (SCA), °**			
Preoperative	2.7 (1.5, 4.0)	2.1 (0.9, 3.4)	0.220
Postoperative 1 year	1.2 (0.5, 2.0)	1.9 (0.7, 3.1)	0.131
Δ† (Post − Pre)	−1.3 (−2.5, −0.5)	0.0 (−0.6, 0.4)	<0.001 **
*p*-value (Pre vs. Post)	<0.001 **	0.936	
**Lumbar lordosis (LL), °**			
Preoperative	46.2 (35.0, 53.1)	43.1 (35.0, 53.1)	0.803
Postoperative 1 year	44.9 (39.1, 54.2)	41.7 (34.6, 51.0)	0.443
Δ† (Post − Pre)	1.6 (−2.7, 6.0)	0.1 (−3.9, 4.0)	0.351
*p*-value (Pre vs. Post)	0.221	0.993	
**PI-LL mismatch, °**			
Preoperative	14.3 (6.8, 20.7)	11.2 (3.5, 22.0)	0.753
Postoperative 1 year	12.2 (6.0, 17.0)	12.6 (1.7, 23.5)	0.859
*p*-value (Pre vs. Post)	0.210	0.755	
Fusion rate	30 (93.8%)	31 (93.9%)	0.974

† Difference between “Preoperative” and “Postoperative 1 year”. **°**, Degree. * *p* < 0.05, ** *p* < 0.01. OLIF, oblique lumbar interbody fusion; TLIF, transforaminal lumbar interbody fusion; PI, pelvic incidence.

**Table 5:** Outcomes for OLIF with (OLIF + D) or without posterior decompression (OLIF − D) and the TLIF group.

	OLIF − D (n = 14)	OLIF + D (n = 18)	TLIF Group (n = 33)	*p*-Value
Age, years	66.0 (58.0, 73.3)	69.0 (62.0, 76.3)	71.0 (66.0, 76.5)	0.366
Female	12 (85.7%)	13 (72.2%)	22 (66.7%)	0.418
BMI, kg/m^2^	27.4 (24.3, 30.3)	26.7 (24.3, 29.5)	26.8 (24.6, 30.8)	0.739
Hospital stay, days	6.0 (5.0, 6.0)	7.0 (6.0, 8.3)	7.0 (6.0, 8.0)	0.007 **
Operating time, mins	295.0 (266.3, 328.8)	362.0 (315.0, 401.3)	300.0 (277.5, 320.0)	0.001 **
Blood loss, mL	270.0 (137.5, 457.5)	400.0 (250.0, 600.0)	600.0 (450.0, 765.0)	<0.001 **
Cage height, mm	12.0 (10.0, 12.0)	8.0 (8.0, 10.0)	10.0 (8.0, 10.0)	<0.001 **
Subsidence	2 (14.3%)	3 (16.7%)	5 (15.2%)	1.000
Reoperation	1 (7.1%)	2 (11.1%)	2 (6.1%)	0.834
**VAS (back pain)**				
Preoperative	8.0 (6.0, 9.0)	8.0 (7.0, 8.0)	8.0 (5.0, 10.0)	0.960
Postoperative 3 months	3.5 (1.5, 5.0)	3.0 (0.0, 3.0)	3.0 (1.5, 4.0)	0.450
Last follow-up	3.5 (0.0, 5.3)	3.0 (0.0, 4.0)	3.0 (2.0, 4.0)	0.584
**VAS (leg pain)**				
Preoperative	8.0 (3.0, 8.0)	7.5 (6.8, 8.3)	8.0 (8.0, 10.0)	0.072
Postoperative 3 months	1.0 (0.0, 3.5)	2.0 (0.0, 5.3)	3.0 (0.0, 3.5)	0.588
Last follow-up	1.5 (0.0, 3.0)	0.0 (0.0, 4.0)	2.0 (0.0, 4.0)	0.576
**Oswestry Disability Index (ODI)**				
Preoperative	58.9 (51.1, 67.2)	64.4 (56.7, 67.2)	60.0 (52.2, 67.8)	0.729
Postoperative 3 months	44.4 (38.9, 54.4)	45.6 (37.2, 52.8)	40.0 (34.4, 53.3)	0.737
Last follow-up	47.8 (26.1, 57.8)	40.0 (28.9, 53.9)	40.0 (30.0, 50.0)	0.474
**EQ5D**				
Preoperative	0.36 (0.36, 0.36)	0.36 (0.36, 0.36)	0.36 (0.36, 0.36)	0.606
Postoperative 3 months	0.60 (0.54, 0.69)	0.60 (0.54, 0.68)	0.60 (0.54, 0.63)	0.907
Last follow-up	0.54 (0.54, 0.83)	0.68 (0.58, 0.72)	0.59 (0.54, 0.71)	0.326

OLIF − D, oblique lumbar interbody fusion without posterior decompression; OLIF + D, oblique lumbar interbody fusion with posterior decompression; TLIF, transforaminal lumbar interbody fusion. ** *p* < 0.01.

## Data Availability

Data and materials are available from the corresponding author on reasonable request.

## Supplementary Materials

The following supporting information can be downloaded at: https://www.mdpi.com/article/10.3390/jcm13195843/s1, Table S1: MCID achievement among surgical group; Table S2: Perioperative data on the OLIF and TLIF groups (Power analysis); Table S3: Outcomes for OLIF with (OLIF+D) or without posterior decompression (OLIF−D) and the TLIF group (Power analysis); Table S4: Radiologic outcomes for the OLIF and TLIF groups (Power analysis). References [32,33].

## Appendix A

The post hoc power analysis, the minimal clinically important differences (MCIDs) in reported outcome measures for each patient, and the proportion of patients who achieved MCIDs in both groups are provided in the Supplementary File.
