# Supplementary material for: A Comparative Study between Single-Level Oblique Lumbar Interbody Fusion with Transforaminal Lumbar Interbody Fusion for Lumbar Adjacent Segment Disease
Source: J Clin Med. 2024 Sep 30;13(19):5843. doi: 10.3390/jcm13195843 (PMC11476864; doi:10.3390/jcm13195843)

Minimal clinically important difference (MCID)

A clinically relevant successful treatment for lumbar interbody fusion surgery was defined as one that achieved a predetermined cutoff value for minimal clinically important difference (MCID). A reduction of **20** in ODI, **2.5** in VAS-Back, **3.5** in VAS Leg, and **0.3** in EQ-5D were considered to be acceptable MCID values for the individual [1, 2]. The proportion of patients who achieved MCID was measured in both groups (**Table S1**).

Table S1. MCID achievement among surgical groups

	**OLIF group (n=32)**	**TLIF group (n=33)**	P-value
VAS (back pain)	75% (n=24)	72.7% (n=24)	0.834
VAS (leg pain)	71.9% (n=23)	75.8% (n=25)	0.721
Oswestry Disability Index (ODI)	59.3% (n=19)	60.6% (n=20)	0.919
EQ5D	56.3% (n=18)	54.5% (n=18)	0.890

OLIF, oblique lumbar interbody fusion. TLIF, transforaminal lumbar interbody fusion.

1. Salaffi, F.; Stancati, A.; Silvestri, C.A.; Ciapetti, A.; Grassi, W. Minimal clinically important changes in chronic musculoskeletal pain intensity measured on a numerical rating scale. *Eur J Pain.* **2004**, *8*, 283-291.
2. Solberg, T.; Johnsen, L.G.; Nygaard Ø , P.; Grotle, M. Can we define success criteria for lumbar disc surgery? : estimates for a substantial amount of improvement in core outcome measures. *Acta Orthop.* **2013**, *84*, 196-201.

Post hoc power analysis

The results of post hoc power analyses for key statistically significant variables are listed below. Post hoc analyses were not conducted for non-significant variables

Table S2. Perioperative data on the OLIF and TLIF groups.

	OLIF group (n=32)		TLIF group (n=33)		*p* value
Length of hospital stay, days	6.0	(6.0, 7.0)	7.0	(6.0, 8.0)	0.172
Operation time, mins	327.5	(292.5, 377.5)	300.0	(277.5, 320.0)	**0.039***
Estimated blood loss, mL	325.0	(231.3, 500.0)	600.0	(450.0, 765.0)	**<0.001****

*P < .05, **P < .01. OLIF, oblique lumbar interbody fusion. TLIF, transforaminal lumbar interbody fusion.

A. Operation time, power=0.52

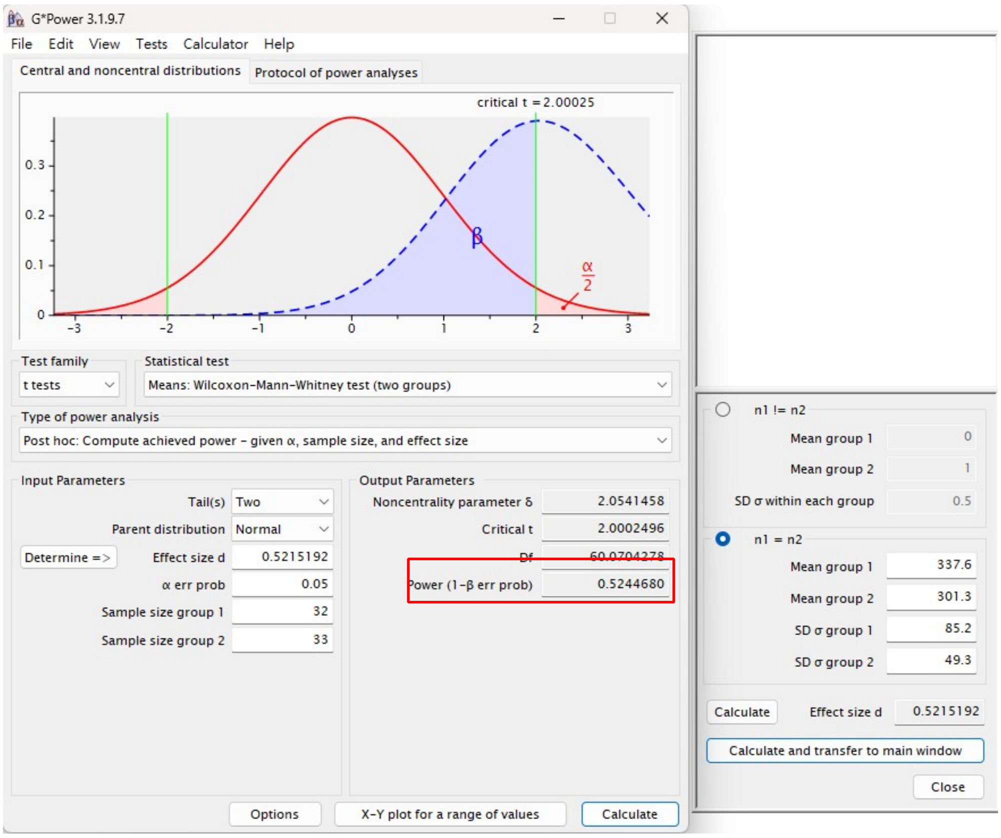

B. Estimated blood loss, power=0.98

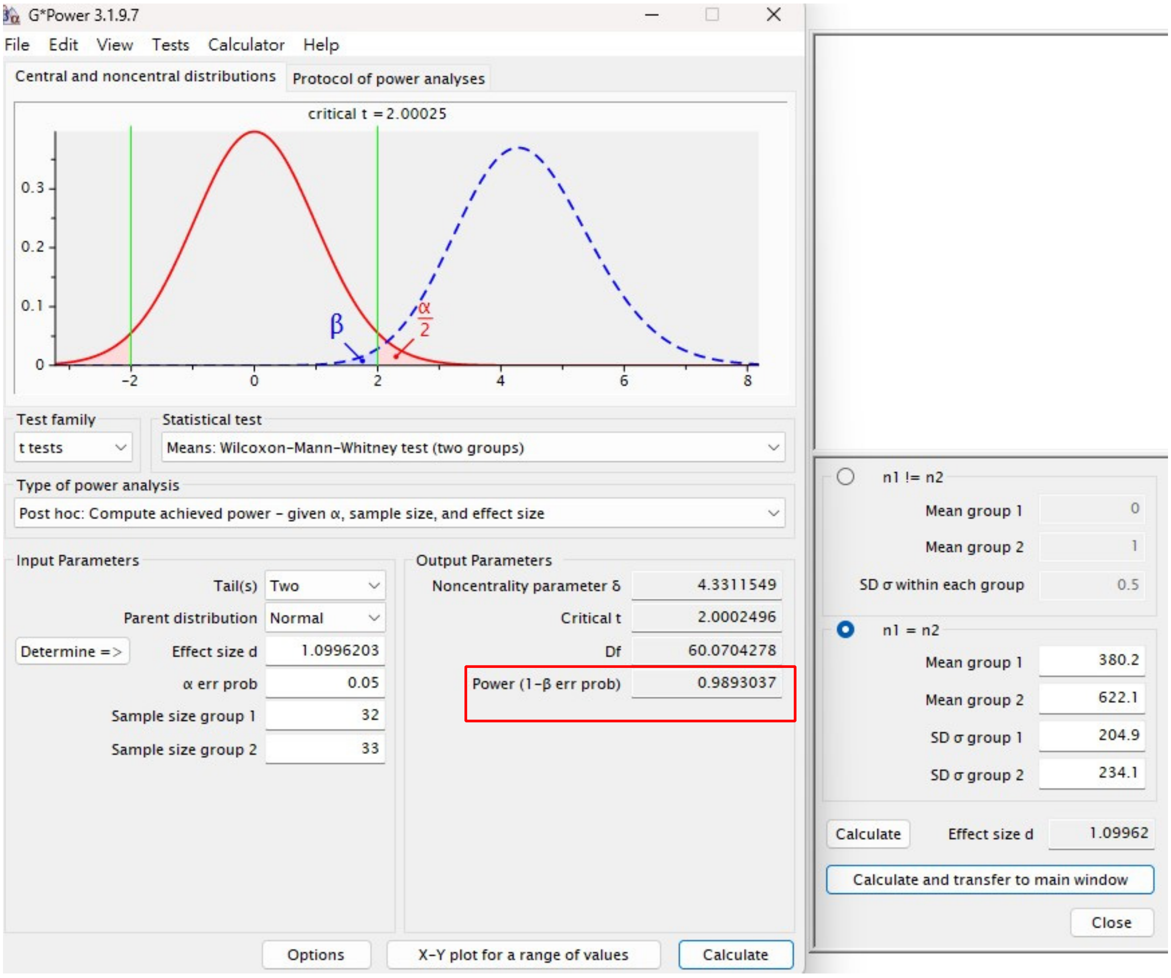

Table S3. Outcomes for OLIF with (OLIF+D) or without posterior decompression (OLIF−D) and the TLIF group.

	**OLIF-D (n=14)**	**OLIF+D (n=18)**	**TLIF group (n=33)**	P-value
Hospital stay, days	6.0 (5.0, 6.0)	7.0 (6.0, 8.3)	7.0 (6.0, 8.0)	**0.007****
Operating time, mins	295.0 (266.3, 328.8)	362.0 (315.0, 401.3)	300.0 (277.5, 320.0)	**0.001****
Blood loss, mL	270.0 (137.5, 457.5)	400.0 (250.0, 600.0)	600.0 (450.0, 765.0)	**<0.001****

*P < .05, **P < .01. OLIF, oblique lumbar interbody fusion. TLIF, transforaminal lumbar interbody fusion. **A.**

Length of hospital stay, power=0.07

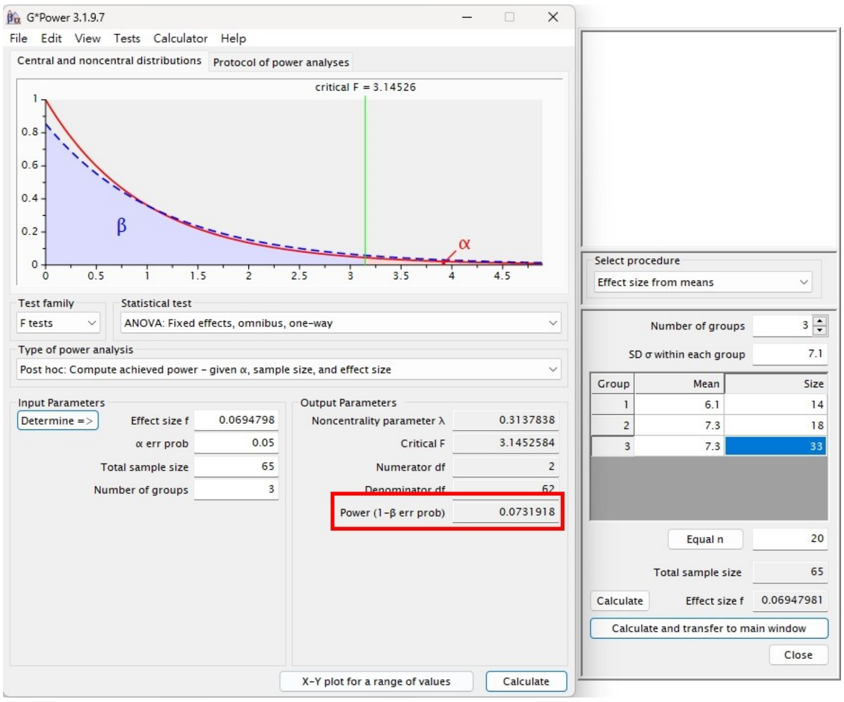

B. Operation time, power=0.93

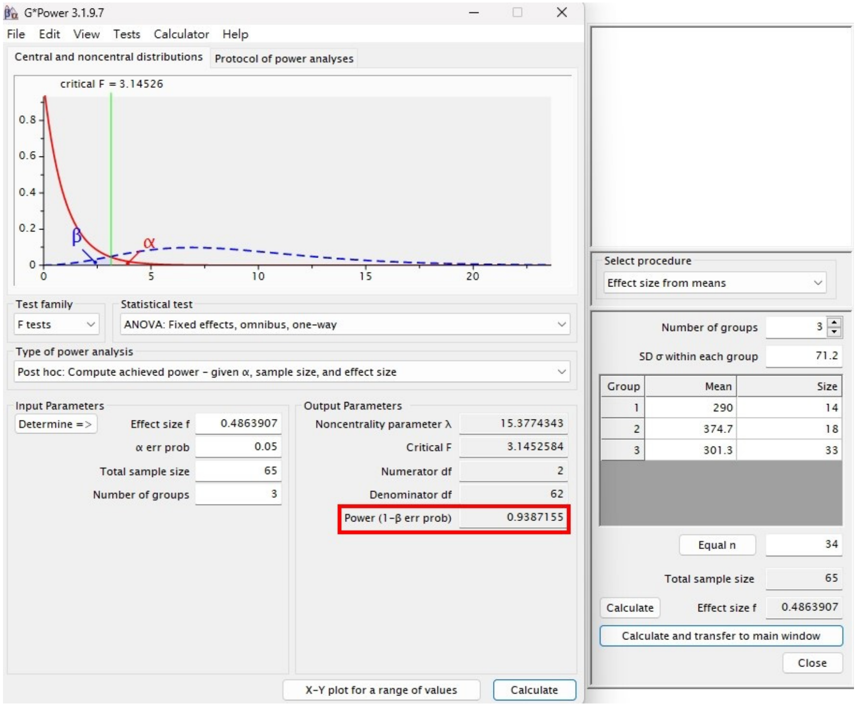

C. Estimated blood loss, power=0.96

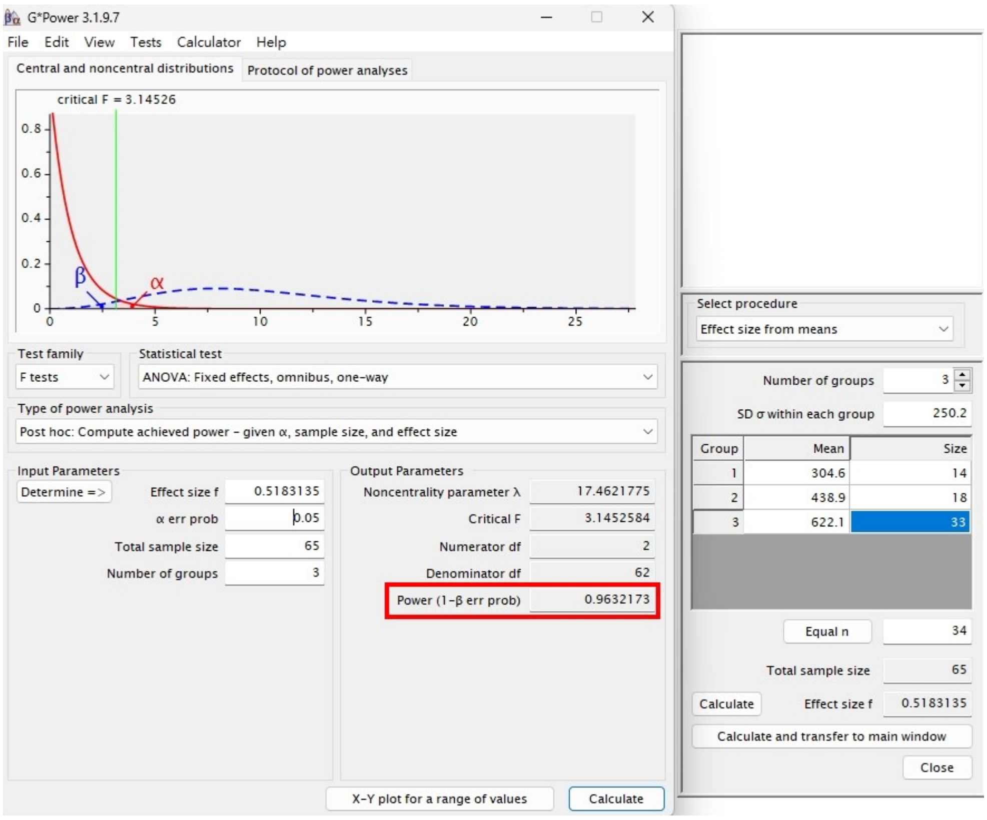

Table S4. Radiologic outcomes for the OLIF and TLIF groups.

	OLIF group (n=32)	TLIF group (n=33)	P-value
**Disc height (DH), mm**			
Preoperative	4.8 (3.9, 7.2)	5.0 (4.0, 6.0)	0.529
Postoperative 1 year	8.8 (7.7, 9.9)	7.1 (6.1, 7.7)	**<0.001****
Δ† (Post - Pre)	2.8 (2.0, 5.0)	2.0 (0.4, 3.5)	**0.011***
**Segmental lordosis (SL), °**			
Preoperative	4.9 (2.4, 7.6)	5.6 (2.2, 9.9)	0.665
Postoperative 1 year	8.5 (7.3, 11.2)	7.0 (4.8, 10.2)	0.065
Δ† (Post - Pre)	3.7 (1.5, 5.5)	1.2 (-1.4, 4.3)	**0.012***
**Segmental coronal angle (SCA), °**			
Preoperative	2.7 (1.5, 4.0)	2.1 (0.9, 3.4)	0.220
Postoperative 1 year	1.2 (0.5, 2.0)	1.9 (0.7, 3.1)	0.131
Δ† (Post - Pre)	-1.3 (-2.5, -0.5)	0.0 (-0.6, 0.4)	**<0.001****
**Lumbar lordosis (LL), °**			
Preoperative	46.2 (35.0, 53.1)	43.1 (35.0, 53.1)	0.803
Postoperative 1 year	44.9 (39.1, 54.2)	41.7 (34.6, 51.0)	0.443

Δ† (Post - Pre)	1.6 (-2.7, 6.0)	0.1 (-3.9, 4.0)	0.351
**PI – LL mismatch, °**			
Preoperative	14.3 (6.8, 20.7)	11.2 (3.5, 22.0)	0.753
Postoperative 1 year	12.2 (6.0, 17.0)	12.6 (1.7, 23.5)	0.859

† Difference between “Preoperative” and “Postoperative 1 year.” *P < .05, **P < .01. OLIF, oblique lumbar interbody fusion. TLIF, transforaminal lumbar interbody fusion. PI, pelvic incidence.

A. Disc height, postoperative 1 year, power=0.99

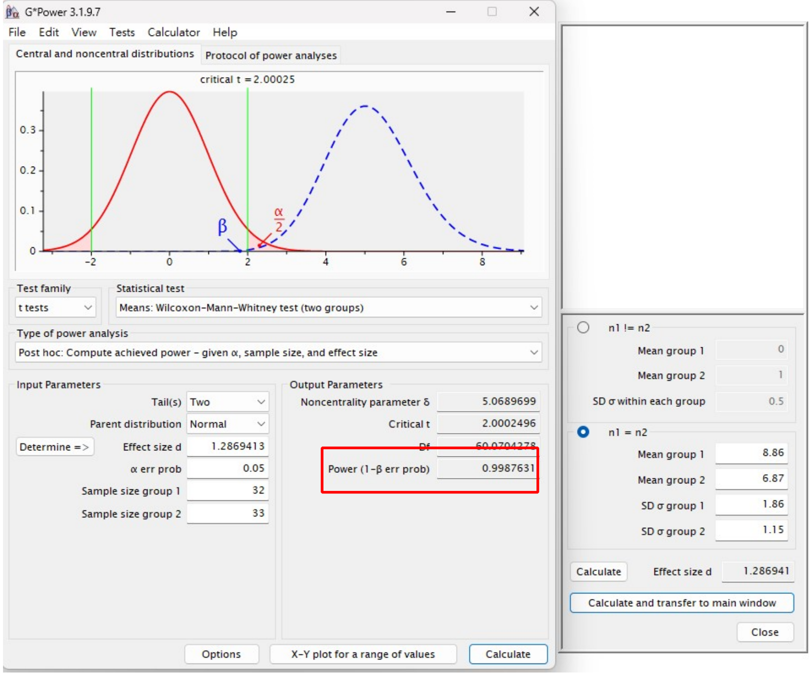

B. Disc height, Δ† (Post - Pre), power=0.85

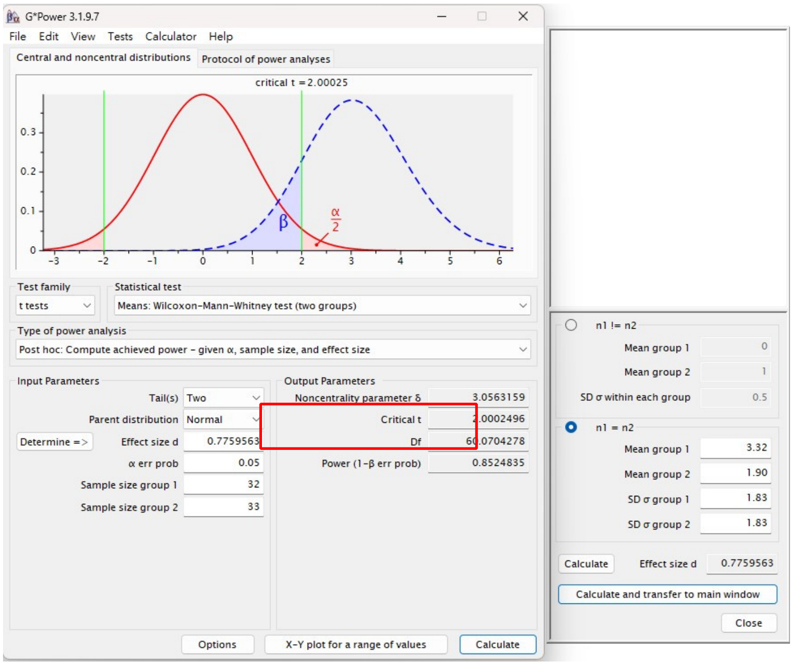

C. Segmental lordosis, Δ† (Post - Pre), power=0.41

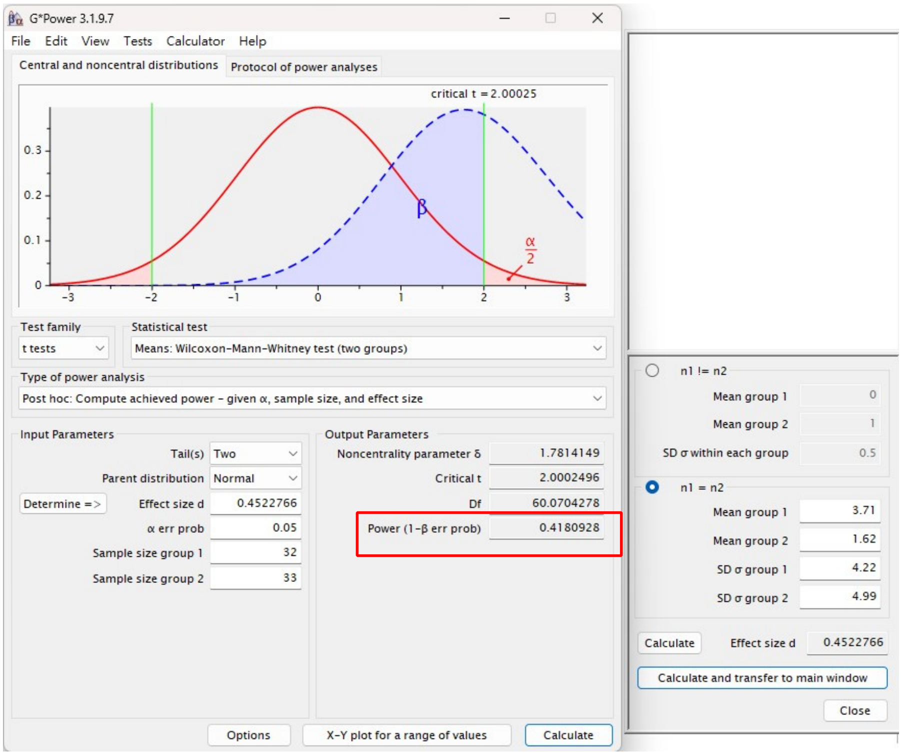

D. Segmental coronal angle, Δ† (Post - Pre), power=0.92

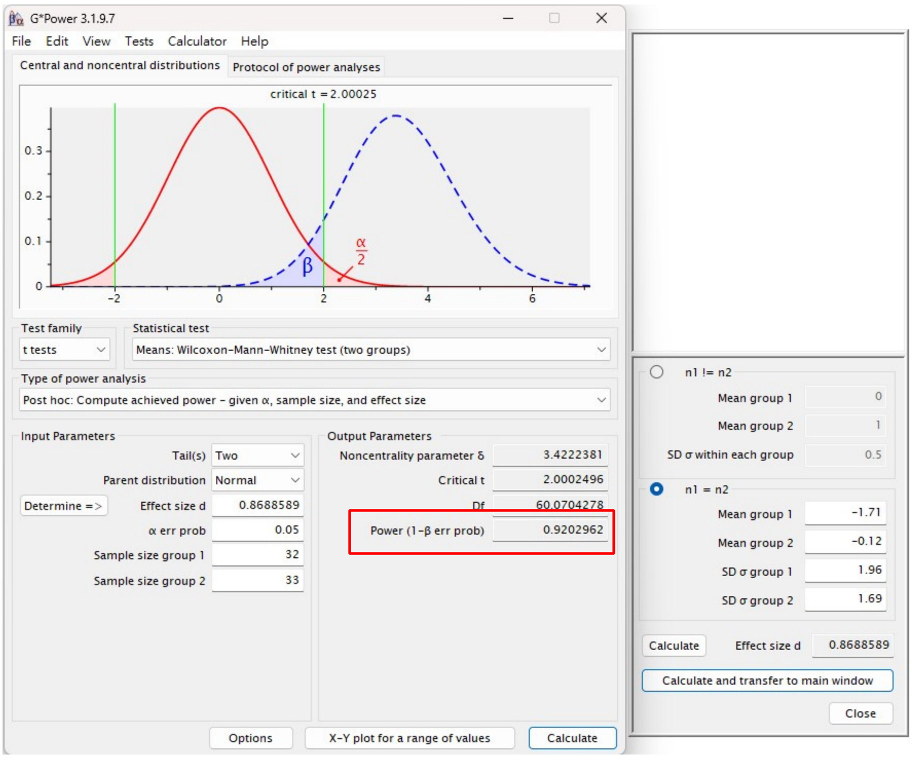

Supplement: Supplementary file 1 [file jcm-13-05843-s001.zip › jcm-3174324-supplementary.pdf]
